# Stimulus Driven Single Unit Activity From Micro-Electrocorticography

**DOI:** 10.3389/fnins.2020.00055

**Published:** 2020-02-28

**Authors:** John Hermiz, Lorraine Hossain, Ezequiel M. Arneodo, Mehran Ganji, Nicholas Rogers, Nasim Vahidi, Eric Halgren, Timothy Q. Gentner, Shadi A. Dayeh, Vikash Gilja

**Affiliations:** ^1^Department of Electrical and Computer Engineering, University of California, San Diego, La Jolla, CA, United States; ^2^Department of Materials Science and Engineering, University of California, San Diego, La Jolla, CA, United States; ^3^Biocircuits Institute, University of California, San Diego, La Jolla, CA, United States; ^4^Department of Physics, University of California, San Diego, La Jolla, CA, United States; ^5^Department of Radiology, University of California, San Diego, La Jolla, CA, United States; ^6^Department of Neurosciences, University of California, San Diego, La Jolla, CA, United States; ^7^Department of Psychology, University of California, San Diego, La Jolla, CA, United States; ^8^Kavli Institute for Brain and Mind, La Jolla, CA, United States; ^9^Neurobiology Section, University of California, San Diego, La Jolla, CA, United States; ^10^Department of Nanoengineering, University of California, San Diego, La Jolla, CA, United States

**Keywords:** electrocorticogram, brain machine interface, neural interface, birdsong, action potential

## Abstract

High-fidelity measurements of neural activity can enable advancements in our understanding of the neural basis of complex behaviors such as speech, audition, and language, and are critical for developing neural prostheses that address impairments to these abilities due to disease or injury. We develop a novel high resolution, thin-film micro-electrocorticography (micro-ECoG) array that enables high-fidelity surface measurements of neural activity from songbirds, a well-established animal model for studying speech behavior. With this device, we provide the first demonstration of sensory-evoked modulation of surface-recorded single unit responses. We establish that single unit activity is consistently sensed from micro-ECoG electrodes over the surface of sensorimotor nucleus HVC (used as a proper name) in anesthetized European starlings, and validate responses with correlated firing in single units recorded simultaneously at surface and depth. The results establish a platform for high-fidelity recording from the surface of subcortical structures that will accelerate neurophysiological studies, and development of novel electrode arrays and neural prostheses.

## Introduction

Songbirds (Oscines) are a critical animal model for studying the neural basis of speech and auditory process, as their songs share many common features with human speech and language (Brainard and Doupe, 2002; Nottebohm, 2005; Bolhuis and Gahr, 2006; Pfenning et al., 2014). Like human speech, birdsong is a learned behavior and can possess a complex temporal and compositional structure. Additionally, the biomechanics of vocal production and vocal anatomy of songbirds have similarities with humans and some non-human primates (Titze, 1988; Gardner et al., 2001; Takahashi et al., 2015). Advances in our understanding of how neural circuits give rise to these complex vocal behaviors are enabled by high-resolution and high-fidelity observations of neural activity. Such views of neural activity can also enable brain-machine interface studies in songbird, providing a path for rapid development and validation of cortically driven speech prosthesis prototypes for individuals with speech and motor impairments.

Recently, micron scale electrocorticography (micro-ECoG) has emerged as a promising tool for recording and stimulating the brain. Given that these electrodes do not require penetrating the brain, they provide the ability to achieve wide spatial coverage while minimizing perturbation of brain tissue. Technological innovations in electrode fabrication have given rise to thin-film electrodes, which further reduce the volume occupied by these electrode arrays and result in electrodes that intimately conform to the surface of the brain (Khodagholy et al., 2011, 2015, 2016; Ganji et al., 2018). The planar fabrication process used to build these devices allows for high density electrode arrays arranged in arbitrary configurations and with arbitrary contact geometry at the micron scale. With contact diameters reduced to 10 s of microns, these arrays permit focal recording but can also result in larger impedance and, consequently, increased measurement noise that may degrade the ability to sense neural signals (Lempka et al., 2011). Fortunately, advances in electrode materials and coatings have improved the electrical properties of these devices, allowing contact size to be scaled down without compromising the ability to record physiological signals. One promising coating is Poly(3,4-ethylenedioxythiophene)-poly(styrenesulfonate) (PEDOT:PSS), an organic polymer that can be spin-cast onto electrodes to greatly reduce impedance, enhancing the ability of electrodes to record neural activity and to stimulate the brain (Khodagholy et al., 2011; Ganji et al., 2017a, b, 2018).

Micro-electrocorticography can complement other neural interface technologies such as penetrating electrode arrays (PEA). For example, penetrating laminar shank style probes provide excellent resolution and are commonly used in neurophysiology studies (e.g., Fukushima et al., 2015; Kozlov and Gentner, 2016; Vyssotski et al., 2016), but lack broad spatial coverage. These PEAs, which can sample spatially at varying depth, could be combined with micro-ECoG electrode arrays that have broad coverage over the surface of the brain to gain new insights into neural dynamics as well as the physiological origin of local field potentials sensed at the surface (Suzuki and Larkum, 2017; Konerding et al., 2018). Micro-ECoG can also provide high spatial resolution. For example, integrated signal power in the 70–110 Hz band recorded from sub-millimeter pitch micro-ECoG devices implanted on human cortex provides significantly more information about brain state than recordings from more coarsely spaced grids (Hermiz et al., 2018). Demonstrations in clinical and rodent experiments provide a proof of concept that micro-ECoG devices have the potential to record single unit activity from the surface of cortex (Khodagholy et al., 2015, 2016).

**FIGURE 1:**
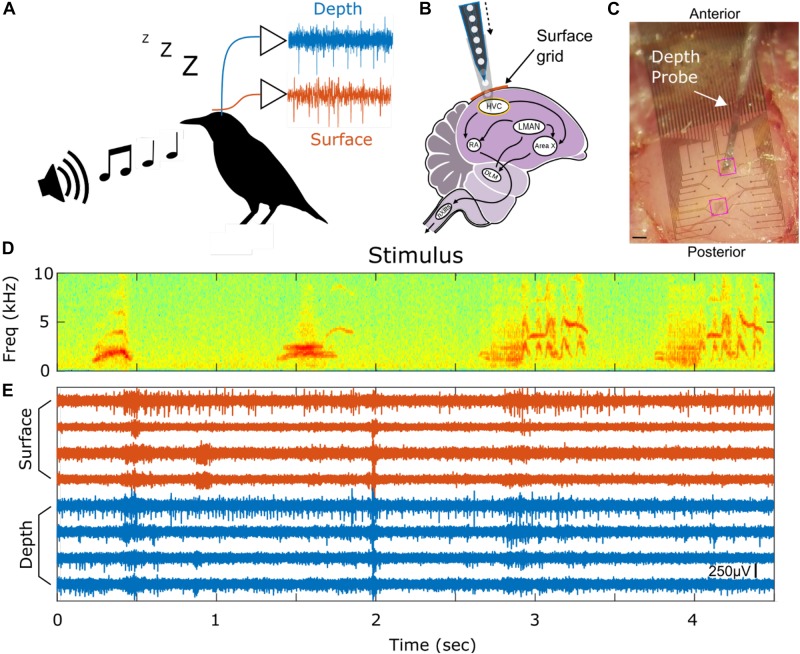
Experimental paradigm and example spiking activity. **(A)** Auditory stimuli are presented to anesthetized European Starlings while extracellular voltage waveforms are recorded simultaneously from surface (red) and depth (blue) probes. **(B)** The micro-ECoG surface array is placed over HVC and the PEA depth probe is inserted into HVC. HVC is at the top of the vocal production pathways as shown in the schematic of the songbird circuit (HVC, used as proper name; RA, robust nucleus of the archipallium; lMAN, lateral magnocellular nucleus of the anterior nidopallium; Area X, used as proper name; DLM, dorsolateral medial thalamus; nXIIts, tracheosyringeal division of 12th cranial nerve). **(C)** Picture of surface grid placed on top of HVC and a depth probe penetrating into the brain through the surface grid. Highlighted in pink rectangles are the two holes where the depth probe can be inserted. Scale bar is 200 μm. **(D)** Stimulus spectrogram showing a short portion of a bird’s-own-song. **(E)** Eight high-pass filtered time series from 4 surface (red) and 4 depth (blue) electrodes showing simultaneously recorded spiking activity. The amplitude scale bar is 250 μV and is located in the bottom right of the figure. onset of the auditory stimulus.

Here, we implant a PEDOT:PSS coated micro-ECoG array over premotor nucleus HVC (used as a proper name) in anesthetized European starlings (*Sturnus vulgaris*, Figure 1A), and observe strong, reliable spiking responses, presumably tied to single neurons. To validate that we are sensing single unit activity (SUA) from the surface of HVC, we present subjects with potent auditory stimuli, namely the bird’s-own-song (BOS) which is known to evoke strong responses in many HVC neurons (George et al., 2005a, b), while recording simultaneously from laminar PEAs implanted in HVC below the micro-ECoG array (Figures 1B,C). This recording configuration enables conventional depth recording of neural units that are approximately 150 μm to 2 mm away from the surface recording sites. The simultaneous surface- and the depth-recorded SUA are driven reliably, and in a correlated manner, by the presentation of BOS (Figures 1D,E).

## Materials and Methods

### Probes

The micro-ECoG electrode array was manufactured in the nano3 facility at UC San Diego using microfabrication techniques similar to those described in Ganji et al. (2018) and summarized in Supplementary Figure S3. The array consists of a 2.9 μm thin Parylene C substrate, gold interconnects and electrodes coated with PEDOT:PSS, resulting in a total thickness of ∼4.2-5 μm. The electrodes have a diameter of 20 μm and are arranged in a grid consisting of 32 electrodes with a 200 μm pitch as shown in Figure 1C and Supplementary Figure S3B. Functional electrodes usually have an impedance around 75 kΩ at 1 kHz as measured in a saline bath. Electrodes with impedances greater than 500 kΩ are deemed not functional. The surface probe has two square holes to allow for a penetrating depth probe to be inserted in between surface electrodes (Figure 1C).

The depth probe is a commercially available silicon shank manufactured by NeuroNexus (Ann Arbor, MI, United States). One of three versions were used throughout the experiments: 16 site probe with 50 μm spacing (A1 × 16-5 mm-50-177-A16) (birds b114, b1061), 32 site probe with three columns of electrodes staggered with an electrode spacing of 25 μm (A1 × 32-Poly3-5mm-25s-177-A32) (bird b1047), and 32 site probe with a linear array of electrodes spaced 20 μm (A1 × 32-Edge-5mm-20_177-A32) (birds b1107, 1067, 1159). The contact area of the 16 and 32 site probes is 177 μm^2^. Functional electrode impedance was typically 1–2 MΩ as measured in a saline bath.

### Subject Selection and Stimuli Generation

Surgical and behavioral procedures were reviewed and approved by the UCSD Institutional Animal Care and Use Committee (IACUC). Acute experiments were performed on anesthetized European starlings, which typically weigh 55–102 g and are 21.6 cm long. In order to induce singing, testosterone was implanted subcutaneously. A 1–3 mm pellet was prepared by filling a segment of silicone tube (Silastic tubing 508-009) with Testosterone propionate solid (Sigma Aldrich T1875-5G) and sealing it at the ends with thick superglue. The animal was anesthetized using Isoflurane, and the pellet was implanted under the skin, through a small aperture achieved with the aid of scissors and a blunt instrument. The aperture was then closed by suture.

Animals were individually housed in a sound-isolation chamber in which audio was continuously recorded via a microphone (Earthworks M30) connected to a preamplifier (ART Tube MP), sampled at 48 kHZ and digitized by the soundcard of a PC using custom software built around the ALSA libraries. Presence of song bouts was automatically monitored nightly from the day’s recordings using custom software written in Python. Birds that would start singing tens to hundreds of bouts a day within the 10 days following implantation were selected for the study.

For each bird selected, a few bouts of birds own song (BOS) were selected, of about 40–60 s each. Stimuli presented included the following: (1) BOS; (2) BOS played in reverse (REV), in which the temporal structure of individual syllables and the global syllable order were reversed but overall spectrum was the same as the BOS; (3) song from a conspecific adult (CON). Several (30–60) presentations of each stimulus of choice were presented at intervals picked from a pseudo-random uniform distribution between 7–15 s, with pseudo-random order within the session.

### Surgical Preparation

Preparatory surgeries were conducted either the day before or the day of electrophysiological recording. Animals were anesthetized with isoflurane (Baxter Healthcare). The birds were head-fixed in a stereotaxic device, and the scalp was dissected along the midline. A custom-built, metallic fixation pin was then attached to the caudal part of the bird’s skull with dental cement.

On the days of recordings, an animal was anesthetized with 20% urethane (60–100 μl total; Sigma, St. Louis, MO, United States) administered into the pectoral muscle in 20- to 30-μl aliquots at 30-min intervals. The bird was placed in a sound-attenuating chamber, and its head was immobilized via the head-fixation pin.

### Electrophysiology

A craniotomy and duratomy was performed over HVC following stereotaxic coordinates. The window was centered at 2.5 mm lateral and was large enough to fit the surface micro-ECoG array. The hippocampus on top of HVC was removed by suction. To ensure intimate contact between the surface array and tissue, cerebral spinal fluid was removed from the surface of the brain by suction. The surface array was then placed on top of the brain using a micromanipulator (Narishige MO-10), and the depth probe was slowly lowered into the brain through one of the two via holes. Both hemispheres of the brain were used; whenever the brain tissue was visibly damaged by the procedure, the site was not further used for the experiment.

Electrophysiological recordings from both the surface array and depth probe were performed simultaneously with the same data acquisition system, Intan RHD2000 from Intan Technologies (Los Angeles, CA, United States). The Intan RHD2000 USB Controller was connected to a RHD2116 or RHD2132 headstage that was connected to the depth probe; a separate RHD2164 headstage was connected to a surface probe. The following adapter boards were used to connect the probe to the Intan headstage: a custom Flex Adapter (Hermiz et al., 2016) for the surface probe and a Plexon (Dallas, TX, United States) N2T A32-HST/32V adapter for the depth probe. Recordings were sampled at either 20 kHz or 30 kHz and data was acquired using either the Open Ephys GUI (Siegle et al., 2017) or RHD2000 software provided by Intan. Default Intan filter settings were used with cutoffs set at 0.01 Hz and 7.5 kHz for data acquisition.

Stimuli were played using software written in Python, running on a single board computer (SBC) (Beaglebone Black). Synchronization with the recording system and later identification of the metadata of the stimuli was achieved by digital trigger pins and/or messages passed using the ZMQ library between the SBC and the Open Ephys recording software. To enable high precision of stimulus onset detection in the recordings, the stimuli were stereo, with one channel containing a 1–5 kHz waveform that was recorded by the Intan system at the same sampling rate as the neural data. (The software is available on https://github.com/zekearneodo/ephysflow/tree/master/rig_tools).

### Spike Sorting and Unit Characterization

All recordings were converted to KWD format, an HDF5 based data model for neural data. Data recorded in the Intan recording software RHD format was converted to KWD using custom software written in Python (Software available on https://github.com/zekearneodo/intan2kwik). OpenEphys software directly supplies KWD support. For visualizing high frequency activity, the raw recordings were high pass filtered forward and backward using a 3rd order Butterworth filter with a cutoff frequency of 300 Hz, to create a sharp cutoff without phase distortion, and stored as a separate KWD file. Spike sorting was performed using KiloSort (Pachitariu et al., 2016). The *post hoc* merge algorithm included in the KiloSort software was used after the main KiloSort algorithm assigned spikes to clusters. The clusters were manually verified by inspecting spike snippets, correlograms and principal components space using Phy (Rossant et al., 2016) and custom Matlab scripts. Spike clusters were labeled either single unit (SUA), multi-unit (MUA), noise, or artifact – noise and artifact clusters were thrown out for all analyses. Clusters were deemed to be SUA if a sub-sampling of spike waveforms exhibited features that are stereotypical of action potential waveforms and if all the spikes in that cluster did not have a substantial number of refractory period violations (e.g., little to no spikes 0 to 2 ms after spiking). Clusters were deemed to be MUA if the spike waveform resembled that of an action potential waveform but had a substantial number of refractory period violations. An example of each cluster label is provided in the Supplementary Figure S4.

Aggregate spike waveform and timing statistics were computed over all single units (Table 1). The definition of each statistic is described here: “Duration” is the peak-to-trough interval; “Spike Rate” is the number of spikes that occurred divided by the number of seconds; “Amplitude” is the maximum minus the minimum point in the average waveform; “Trough/Peak” is the ratio of the trough and peak values; “Symmetry” is characterizes similarity in waveform shape about the center of the waveform; “Bursts or Not” characterizes if the unit tends to fire within blocks of time and is determined by the void parameter as described in the section “Materials and Methods” of a previous study focused on characterizing interspike intervals and bursts in neuronal activity (Selinger et al., 2007). Briefly, the void parameter is a statistic based on the distribution of the logarithm inter-spike interval (ISI), which captures short and long scale spike timing. Precisely, the definition of the void parameter is      1-g⁢(m⁢i⁢n⁢i⁢m⁢u⁢m)s⁢q⁢r⁢t⁢(g⁢(p⁢e⁢a⁢k⁢1)⁢g*⁢(p⁢e⁢a⁢k⁢2)), where *g*(.) is distribution of the log ISI.

**TABLE 1:** Single unit characterization and statistics.

	**Surface (*n* = 23)**	**Depth (*n* = 46)**	***P*-Value**	**Test**
*Duration*	0.167 ms	0.5 ms	5.0e-6	Rank sum
*Spike Rate*	1.95 Hz	1.52 Hz	0.41	Rank sum
*Amplitude*	53.4 μV	107.3 μV	4.3e-5	Rank sum
*Trough/Peak*	−0.65	−0.32	7.0e-8	Rank sum
*Symmetry*	−0.73	−0.13	1.9e-4	Rank sum
*Bursts or Not*	14/23 = 61%	34/46 = 74%	0.27	Chi-square

### Cross Correlation Analysis

Cross correlograms between depth and surface spikes were computed for depth and surface spikes. Cross correlograms are computed by counting the number of times neuron Y (surface unit) fired after or before neuron X (depth unit) within 5 ms bins for a range of lags from −100 to 100 ms. Cross correlograms are calculated either for baseline periods only (no auditory stimulus) or for both baseline and auditory stimulus periods.

Spike activity similarity between SUAs was characterized by using SPIKE-distance (Kreuz et al., 2013). SPIKE-distance is a parameter-free method that uses the relative timing between spikes from two spike trains to determine their similarity. This distance metric ranges from 0 to 1. Zero indicates identical or synchronous spike trains, while larger distances indicate dissimilar or increasingly dyssynchronous spike trains. This measure uses both long and short duration relationships between compared spike trains and thus it is applied to contiguous periods of time that includes both baseline and auditory stimulus periods.

### Analysis of Stimulus Evoked Response

For stimulus-based analysis, the spike counts were calculated within 5 ms bins. A smoothed estimate of the average spike count was computed by taking the average across all trials and then smoothing with a 5th order moving average filter. The amplitude envelope of the auditory stimulus was estimated by using the Hilbert transform, low pass filtering and then downsampling to 200 Hz to match the sample rate of 5 ms binned spiking activity.

The delay between the auditory stimulus and neural response was estimated by using the cross correlation. That is, the cross correlation between the average smoothed spike count and the amplitude envelope of the auditory stimulus was computed for lags less than 200 ms – lags greater than 200 ms are assumed to be less physiologically relevant. The peak (in absolute value) lag was determined to be the delay, and the auditory stimulus was shifted forward by this amount so that the neural response and auditory stimulus are coherent.

We decided to focus our analyses on the initial response to the stimulus, as it yielded the largest and most robust neural response. The onset of the first sound was found by manually inspecting the spectrograms of the stimuli. 200 ms prior to the onset and 300 ms after the onset was taken to be the window of interest over which the subsequent metrics were computed.

The Pearson correlation is computed between the average spike count and envelope of the auditory stimulus. Since there are at most 2 auditory stimuli played to the subject per run, the one that yielded the higher correlation was considered. Correlations that had a *p* > 0.01 (Bonferroni corrected) were deemed spurious and not included in the presented analyses. Similarly, only the effect size and the lag of neural responses that were significantly correlated with the auditory stimulus are considered.

In order to quantify the magnitude of the response, a metric we call the effect size was computed. The definition of effect size is: (μ_*peak*_−μ_*base*_)/σ_*base*_, where μ_*peak*_ is the average spike count in 5 ms bins in a window of ± 50 ms about the peak response. μ_*base*_ is the average spike count in 5 ms bins in a baseline window lasting 1 s prior to the stimulus presentation. Finally,σ_*base*_ is the standard deviation of the spike counts in the baseline window.

Unless specified otherwise, all analyses were performed using custom Matlab software (Natick, MA, United States).

## Results

We record neural activity from the surface probe in all six subjects studied. Specifically, in five out of the six subjects, single unit activity (SUA) is detected, whereas multi-unit activity (MUA) is detected in all subjects (see section “Materials and Methods” and Supplementary Material for definition of SUA and MUA and examples). To validate putative surface-recorded SUA, waveform shape and spiking statistics are evaluated, demonstrating characteristics consistent with SUA. Surface-recorded SUAs are shown to be correlated with depth-recorded SUAs; Surface-recorded SUAs are also consistently modulated by the presence of auditory stimulus in a manner that is similar to that of depth-recorded SUAs.

### Comparison of Depth- and Surface-Recorded Single Unit Waveform Characteristics

**FIGURE 2:**
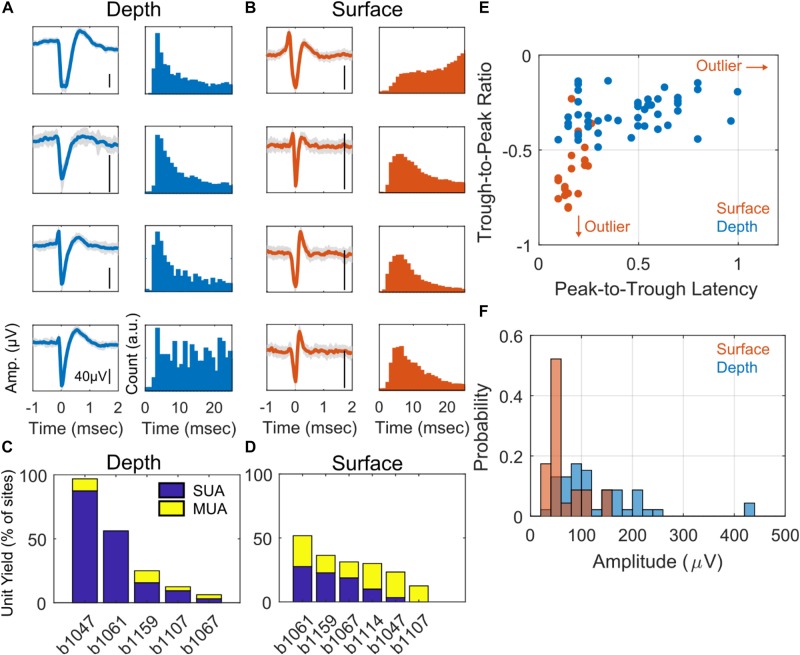
Single unit characteristics. Example of four depth-recorded **(A)** and four surface-recorded **(B)** single unit voltage waveforms and the respective inter-spike interval (ISI) histograms. Waveforms are averaged over 50 uniformly sampled spike events. The *y*-scale bar indicates 40 μV in amplitude. **(C)** Unit yield for depth **(C)** and surface **(D)** arrays as a percentage of total number of contacts. The stacked bar plot shows the percentage of single units (SUA) and multi-units (MUA) for each subject. **(E)** Scatter plot showing trough-to-peak ratio vs. peak-to-trough interval for all putative neurons (units) recorded from the surface (red) and depth arrays (blue), respectively. The red arrows indicate an outlying sample from a surface SUA in the direction that the arrows point. **(F)** Histogram of surface (red) and depth (blue) single unit amplitudes in μV.

Examples of SUA detected on depth and surface probes are shown in Figures 2A,B along with their respective inter-spike interval (ISI). Note that the ISI histograms are consistent with the presence of a refractory period, as we would expect for a single neuron isolation. The unit yield is defined to be the number of channels where there is SUA (or MUA) divided by the total number of functional electrodes (Figures 2C,D); the average SUA yields for surface and depth units are 13.7 and 28.7%, respectively. The depth and surface spike waveforms tend to differ with respect to shape-based features, such as duration and relative trough and peak amplitudes. In the analyses, peak is defined as the minimum point in the waveform (initial depolarization) and the trough is the maximum point that occurs after the peak. Furthermore, duration, or peak-to-trough latency, is defined to be the time between the peak and trough. In general, the depth spikes tend to have a longer duration and larger peak relative to trough than do the surface spikes. This is consistent over the entire dataset of depth and surface SUA as shown in Figure 2E. There is a clear cluster of surface SUA in the lower left portion of the graph, whereas the depth SUA occupies the upper portion of the graph. Furthermore, depth units are more likely to have a larger amplitude than surface units (Figure 2F). Additional spike statistics are computed for depth and surface SUA and are summarized in Table 1. Waveform characteristics (duration, amplitude, trough/peak, symmetry) differ significantly (*p* < 0.01) between depth and surface SUA, although the duration of both surface and depth units is consistent with a previously reported duration range for depth SUA of 0.18–0.85 ms (George et al., 2005b). Spiking characteristics (spike rate and bursting), in contrast, are not significantly different between depth and surface units in this anesthetized experimental setting.

### Correlation Between Putative Surface SUA and Depth SUA

**FIGURE 3:**
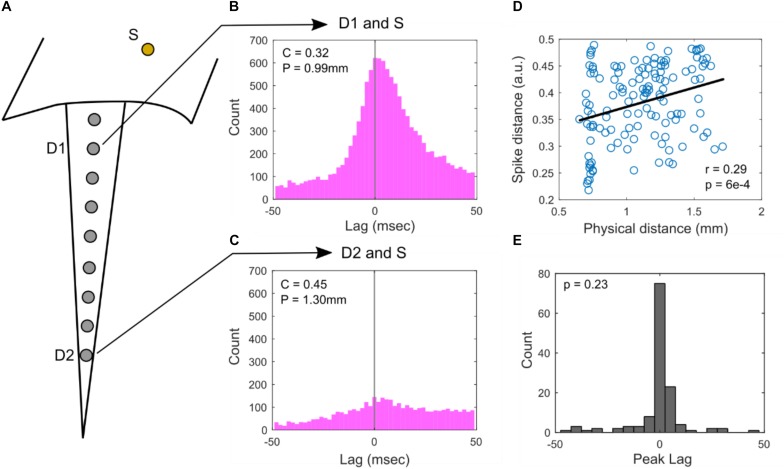
Cross-correlograms. **(A)** Illustration of electrode locations: surface (S) and superficial depth electrode (D1) and deep depth electrode (D2). SUAs were detected on these electrodes and their correlograms were computed in **(B)** and **(C)**. **(B)** Cross correlation of S and D1, showing high co-occurrence. Spike distance, C and physical distance, P are relatively low. **(C)** Cross correlation of S and D2, showing lower co-occurrence. Spike distance, C and physical distance, P are relatively high. **(D)** Population analysis of all surface and depth SUA pairs comparing spike distance vs. physical distance. There is a significant Pearson correlation of *r* = 0.29 (*p* = 6e-4, *n* = 131, Student’s *t*-distribution). **(E)** Histogram of the peak lag appears to be positively skewed; however, the distribution does not significantly deviate from 0, suggesting no significant positive or negative lag between surface and depth units (*p* = 0.23, *n* = 131, rank-sum test).

An analysis across baseline and auditory stimulus periods of cross-correlograms calculated between spike rasters of depth- and surface-recorded single unit pairs is summarized in Figure 3. Figure 3A illustrates how pairs are formed between a depth electrode and a surface electrode. Example cross-correlograms between a single surface electrode and two different depth electrodes are shown in Figures 3B,C. In Figure 3D, the calculated SPIKE-distance of depth and surface spike raster pairs are plotted versus the physical distance between the electrodes that the corresponding units were recorded on. From this plot, we can see that the closest surface and depth electrodes from which SUAs were detected are at least 600 μm apart. Thus, it is highly unlikely for any pair of surface and depth electrodes to be recording from the same neuron. There is a statistically significant positive correlation between the SPIKE-distance measure (Kreuz et al., 2013) and physical distance. SPIKE-distance is 0 for identical spike trains and the measure increases up to a value of 1, which indicates two complete dyssynchronous spike trains. Thus, the positive correlation indicates that as the distance between the electrodes on which depth and surface units increases, their rasters tend to become less synchronous (also see Supplementary Figure S2C for a complementary analysis during only baseline periods). The histogram of peak lag values appears to be positively skewed, suggesting that depth units may tend to precede surface units; however, the distribution does not significantly deviate from 0 ms, indicating no significant average lag between surface and depth units (Figure 3E, also see Supplementary Figure S2D for this analysis during only baseline periods).

### Auditory Stimulus Driven Modulation of Putative Surface SUA and Depth SUA

**FIGURE 4:**
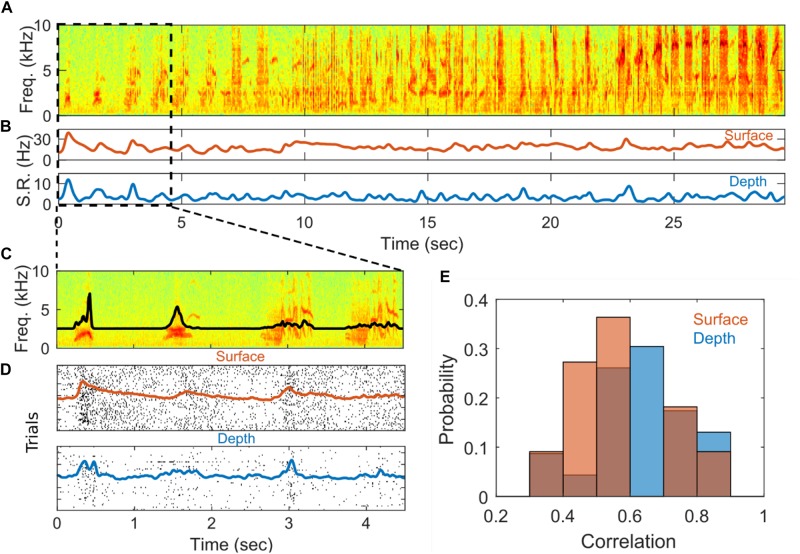
Stimulus driven responses. **(A)** Example spectrogram of a stimulus, Bird’s-own-song presented during electrophysiological recording. **(B)** Averaged smoothed spike rate for single units recorded from the surface (red) and depth (blue) arrays. The average spike rates were smoothed with a 5-point Gaussian window with a standard deviation of 0.4. **(C)** Zoom-in of first 4.5 s of **(A)**. The black line shows the amplitude envelope of the stimulus. **(D)** Spike raster plots showing the times of individual spiking events on each of 54 successive stimulus presentations for the surface (red) and depth (blue) SUAs in **(B)**. The red and blue lines show the smoothed spike rates averaged across stimulus presentations for the surface and depth units, respectively. The spike rates were smoothed with a 30-point Gaussian window a standard deviation of 0.4. **(E)** Distribution of correlation values for depth (blue) and surface (red) for units that are significantly correlated with onset of the auditory stimulus.

The SUA recorded from depth and surface arrays is modulated by auditory stimuli. An example of this modulation from a depth and a surface single unit can be seen in Figures 4A–D. In Figures 4A–C, the spectrogram of a single auditory stimulus presentation, bird’s own song in this example, is plotted alongside the temporally smoothed trial-averaged spike rate for each unit. The spike rate of both the depth- and surface-recorded units appears to be modulated by features of the stimulus, particularly at the beginning of the stimulus. Figures 4C,D zooms in to highlight the first 4.5 s of the stimulus. Panel C is the spectrogram of the stimulus with the stimulus amplitude envelope plotted on top of the spectrogram. Panel D shows spike raster plots with average spike rate plotted on top. Here, a strong response due to the initial sound and a smaller response at 3 s aligned to a more complex vocal element of the birdsong can be observed. When a variant of this complex vocal element reoccurs at around 4 s, it elicits a smaller response. Responses of HVC neurons to natural auditory stimuli, and in particular the BOS, are very well-documented in multiple songbird species (George et al., 2005a; Hermiz et al., 2016; Siegle et al., 2017). Previous reports in starlings show that individual syllables from BOS and conspecific song evoke auditory responses with varying specificity (George et al., 2005a, b). It is well known, at least in other species, that HVC auditory responses can integrate over long time scales and are sensitive to specific temporal (and harmonic) combinations of song elements (Margoliash, 1986; Margoliash and Fortune, 1992).

As a simple test to determine if the recorded SUA is modulated by the auditory stimuli, we characterize the response at the initial onset of each stimulus following a prolonged inter-stimulus interval (7–12 s) during which no stimulus is presented. The initial SUA response is quantified by first computing the Pearson correlation between the average spike count and the amplitude envelope of the stimulus over a 500 ms window centered around the stimulus onset (after correcting for a delay in the neural response, see section “Materials and Methods”). Correlations are deemed spurious if they have an associated *p* > 0.01 (Bonferroni corrected). To quantify the magnitude of the response, an effect size metric is computed for SUA with responses that are significantly correlated with the stimulus. The effect size metric captures the difference in spiking activity between a peak response window and a baseline window and is normalized by variation in the baseline spiking activity (see section “Materials and Methods” for precise definition). Finally, the lag between auditory stimulus and spiking activity is computed for depth and surface. The histogram for depth and surface correlation is in Figure 4E. The distributions of correlation values for depth and surface units appear to be similar. Hypothesis testing also indicates that the distribution of correlation values is not significantly different from each other within the constraints of these data points (*n*_*sur*_ = 11 and *n*_*dep*_ = 23). These statistics are summarized in Supplementary Table S1.

## Discussion

Stimulus modulated SUA recorded from the surface of sensorimotor regions in the songbird brain is presented. Average spike rates from putative surface-recorded neurons show a marked increase during stimulus presentation, particularly at the start of song stimuli. In total, about half of the depth and surface single units are significantly correlated to the amplitude envelope of the initial phase of the stimuli. Stimulus correlation values of surface SUA are comparable to depth SUA and no significant differences in effect size are found.

Raster cross-correlation analyses do not indicate a specific relative timing bias between surface and depth units. However, spike raster similarity analyses indicate that SUA recorded at depth and SUA recorded at surface tend to be more similar if they are recorded at electrodes that a physically closer together. Since surface unit recording locations in this study are at least 600 μm from the closest depth locations, this similarity is very unlikely to be the result of recording the same neural unit from a depth electrode and a surface electrode. Overall, the analyses of spiking characteristics, both stimulus conditioned and not, suggest that the response properties of surface and depth units are similar.

Surface-recorded SUA waveform characteristics appear to be distinct from those of depth-recorded SUAs. In particular, peak-to-trough latency is significantly shorter for surface SUA than depth SUA (Figure 2). Furthermore, the ratio of trough-to-peak amplitudes as well as absolute amplitude appear to be significantly different between surface and depth SUA (Figures 2E,F and Table 1). One possibility is that these differences are due to unmatched filtering characteristics between the depth and surface recordings. As the same amplifiers were used to record from surface and depth, if such a difference exists it would likely be driven by the electrical characteristics of the physical electrode-brain interface. Another possibility is that surface and depth recordings are biased to sample different cell types or different locations on a cell (or both). Waveform shapes difference can be explained by both the biased cell type and location hypotheses (Buzsáki et al., 2012). HVC contains several types of neurons including multiple classes of projection neurons targeting the robust nucleus of the arcopallium (RA) and basal ganglia nucleus Area X, as well as multiple interneurons, who project locally within HVC (Rossant et al., 2016). These different neuron types have different morphologies (Benezra et al., 2018), but the heterogeneity in their spatial distribution (if any) remains mostly unresolved. A previous report indicates that a group of cells with a tendency toward longer or bursting responses is centered in the ventral part of HVC (George et al., 2005a), and this would be consistent with our measurements of a higher ratio of bursting in the cells recorded with the penetrating probe. More detailed understanding of the biases in our surface electrodes for sensing a particular neuron type or its projections remains a topic for future work. In mammalian brain, cortical neuronal organization and morphology have been better characterized, and it may be possible to design electrodes that target specific cell classes.

Low surface area and low impedance electrode contacts, along with the conformality of the arrays to the brain surface, are important characteristics for recording putative action potential activity from the brain surface as demonstrated in this study and in previous mammalian studies (Khodagholy et al., 2015, 2016). In a modeling study (Hill et al., 2018) evaluating critical characteristics for recording action potentials from the cortical surface of the rat, small electrode surface area facilitates retention of action potential amplitude relative to larger electrodes. However, as surface area decreases for an electrode of a given material, the impedance will increase and, consequently, thermal recording noise will also increase. Thus, it is crucial to fabricate small surface area electrode contacts with materials that permit a low impedance electrical interface to the brain surface. We note that our electrodes have over three times the surface area of the electrodes in previous mammalian studies (Khodagholy et al., 2015, 2016). This may have contributed to our lower and variable yield of electrodes with single- and/or multi-unit activity (Figure 2D) relative to previous cortical recordings in rat [55–93% yield as reported in Supplementary Table S1 of Khodagholy et al. (2015)]. However, yield in human recordings (27–37%) (Khodagholy et al., 2015) is closer to our yield. These yields and their variability could be due to a wide range of factors beyond electrode design, including species differences and specifics of surgical and recording procedures. For example, the rodent recordings were conducted while the animal was awake and behaving; while our starling recordings and their human recordings were conducted under anesthesia, potentially influencing the probability of neural spiking.

Electrode array conformality is also of importance for recording quality. According to the modeling study (Hill et al., 2018), neuron cell bodies (in the rat preparation) must be within 60 μm of a 100 μm^2^ electrode contact (our electrodes have a 314 μm^2^ surface area, which should reduce the distance requirement). Furthermore, if electrodes are further from the cortical surface and have cerebral spinal fluid (CSF) under electrode contacts, this could cause shunting that would obscure action potential recording. The modeling study (Hill et al., 2018) also suggests that the conformal insulation of the electrode array substrate around the electrode may also be important for resolving action potentials. Surface insulation effectively creates a reflection of charges that does not exist at the natural conductive CSF interface. Thus, insulation can effectively boost the action potential signal relative to thermal noise. A previous rat study (Tsytsarev et al., 2006) suggested that multi-unit activity could be recorded from the brain surface, but did not demonstrate single-unit like activity. In that study an array of 50 μm diameter microwires were brought to the cortical surface. Beyond the larger surface area relative to the electrodes in the current study, those microwire electrodes have higher impedance and lack the planar insulation of thin film electrode arrays. These design characteristics of the microwire bundle may have resulted in reduced signal quality, obscuring single unit activity. Electrode characteristics at the micro and nano scale provide avenues for future enhancements of recording quality. For example, electrodes in planar arrays for *in vitro* recording preparations can be modified to have a 3D mushroom-like shape, resulting in more intimate contact with cell membranes and higher signal to noise ratio recording (e.g., Spira et al., 2018). Electrode arrays for *in vivo* surface recordings could employ similar design strategies to improve the quality and reproducibility of tissue contact.

While the current study is acute, thin film micro-ECoG preparations have been employed for chronic recordings in multi-day awake behaving chronic preparations in small animal models, including mouse (e.g., Jonak et al., 2018) and in rat (e.g., Insanally et al., 2016). Chronic studies with the electrode arrays used in the current acute study would require additional device and surgical technique development, as well as longevity testing. Such development would allow for richer study of the unit activity to assess stability and response characteristics during free behavior and song production.

In summary, we demonstrate the ability of micro-ECoG electrode arrays coated with PEDOT:PSS on a 4–5 μm thin Parylene C substrate to sense single units over HVC in anesthetized European Starling. These single units are found in five out of six subjects with an average yield of 13.7%. An increase in spiking activity is observed at the onset of the auditory stimulus for both surface and depth single units. Roughly half of the single units for both surface and depth are significantly correlated with the onset of the auditory stimulus. These results reproduce and extend an important finding, which is that single units can be sensed from the surface of the brain, and their activity can be modulated with sensory relevant stimuli. By providing a first demonstration of stimulus driven response from surface recorded units, the results of this study increase confidence that these units are indeed single neurons and that micro-ECoG is an effective tool for observing neural activity with high-fidelity. Furthermore, by demonstrating these capabilities in songbird, we provide a new paradigm for studying the neural basis of speech and language and a development platform for cortically driven speech prostheses.

## Data Availability Statement

The datasets generated for this study are available on request to the corresponding author.

## Ethics Statement

The animal study was reviewed and approved by the UCSD Institutional Animal Care and Use Committee.

## Author Contributions

JH performed the data analysis, assisted with the data collection instrumentation, and co-wrote the manuscript. LH fabricated and characterized the micro-ECoG devices and participated in electrode placement and data collection. EA participated in the design of the experiment, built the experimental setup, led the data collection, contributed tools for data processing/analysis, and provided input to the manuscript. MG participated in micro-ECoG device fabrication and characterization. NR contributed to experiment design aspects related to instrumentation and novel device usage. NV contributed to the conception of the simultaneous surface/depth recording experiments. EH provided guidance for neurophysiological analysis. TG guided experiment design and data collection methods, and co-wrote the manuscript. SD helped to conceive and design the experiments and oversaw the micro-ECG device fabrication and characterization. VG oversaw data analysis, guided experiment design and data collection methods, and co-wrote the manuscript.

## Conflict of Interest

JH is currently employed by Vorso Corp. VG holds shares in Neuralink, Corp., and Paradromics, Inc., and currently consults for Paradromics, Inc. The remaining authors declare that the research was conducted in the absence of any commercial or financial relationships that could be construed as a potential conflict of interest.
