# Supplementary material for: Stimulus Driven Single Unit Activity From Micro-Electrocorticography
Source: Front Neurosci. 2020 Feb 28;14:55. doi: 10.3389/fnins.2020.00055 (PMC7059620; doi:10.3389/fnins.2020.00055)
Supplement: Supplementary file 1 [file Data_Sheet_1.pdf]

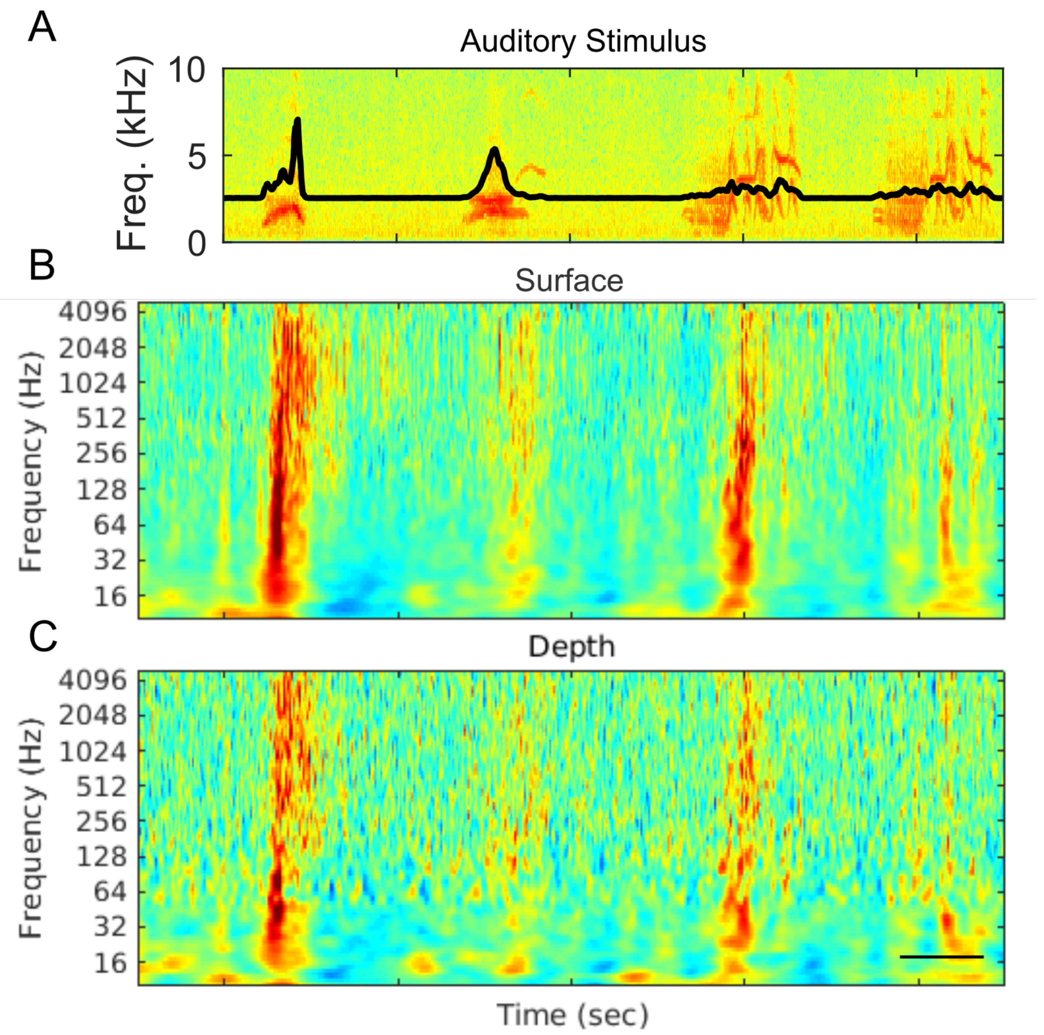

S-Figure 1: Average scaleograms. A) Spectrogram of auditory stimulus (bird's own song). This is the same stimulus example shown throughout the main body. Trial averaged scaleogram of neural response measured from an electrode at B) the surface and from an electrode C) at depth.

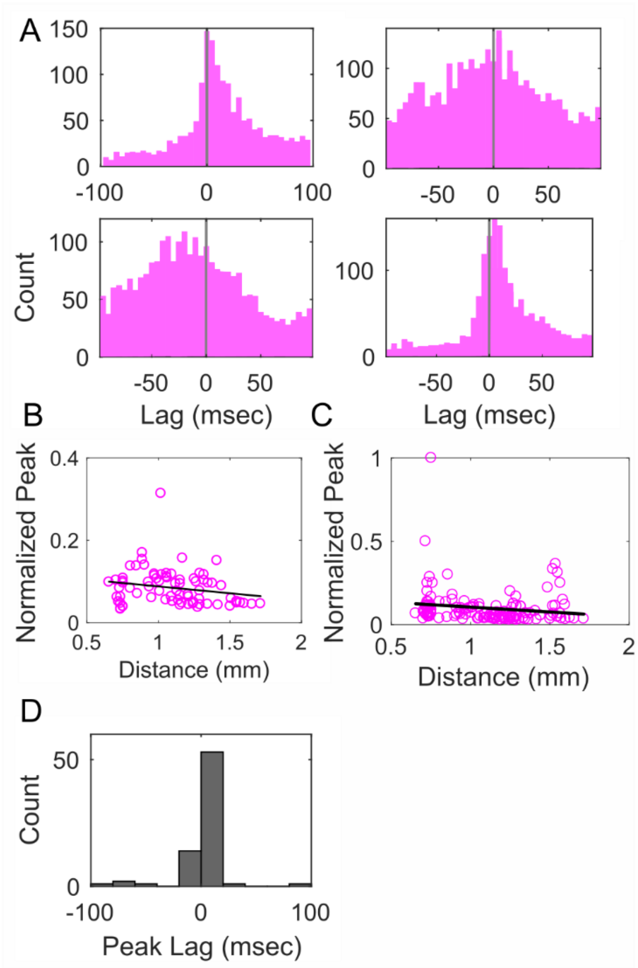

S-Figure 2: **Cross-correlograms.** A) Example cross-correlograms between SUA recorded simultaneously from depth and surface arrays during the baseline periods only (times between auditory stimulation). The lag of 0 is the time at which the depth spike occurred. B) Normalized cross-correlogram peak vs. distance between electrode contacts, calculated from only the baseline preiods. The normalized peak is the fraction of spikes that occur in the peak bin within the window of interest (-100 to 100 msec). A line of best fit suggests that as distance between electrodes increases, the neural units recorded on each of these electrodes tend to have a smaller normalized cross correlogram peak (Pearson: r = -0.20, p = 0.078; Spearman: r = -0.21; n = 75). C) Same as B, but using both baseline and auditory stimulus periods as Figure 3D. Again, there is a weak negative correlation (Pearson: r = -00.15; p = 0.085) D) Histogram of the peak lag calculated from baseline periods appears to be positively skewed; however, the distribution does not significantly deviate from 0, suggesting no timing relationship between surface and depth units (p = 0.81, n = 75).

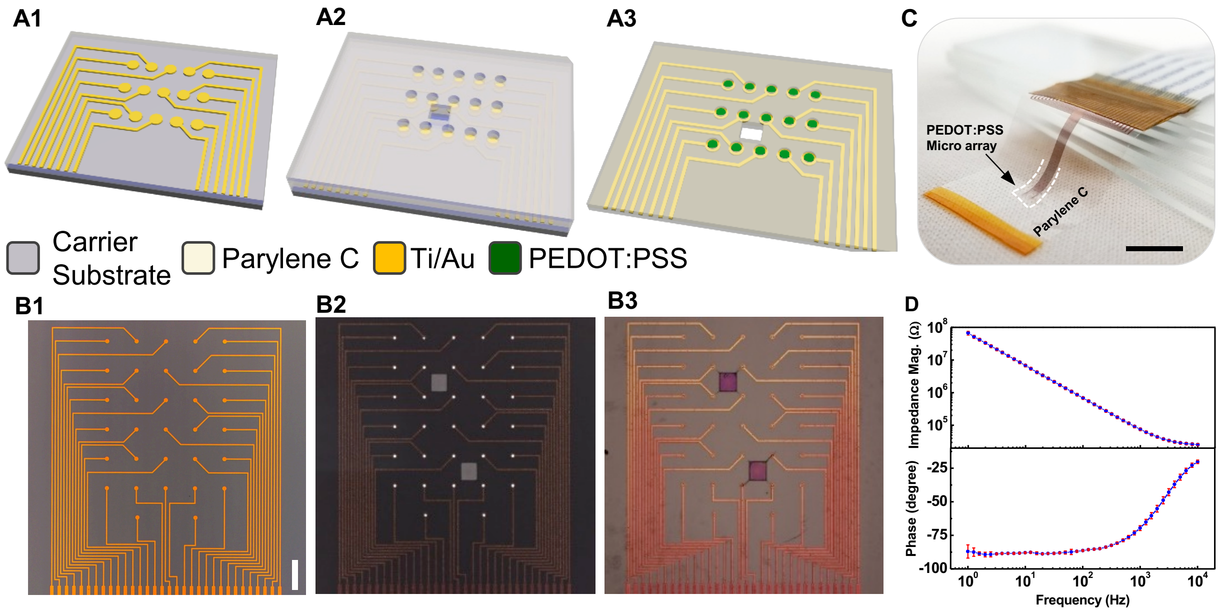

S-Figure 3: Fabrication of the PEDOT:PSS neural device. A) Schematic of monolithic device processing that results in an encapsulated flexible probe. B) Optical images of device after metallization (B1), via etching of the parylene C to expose electrode contact region (B2), and coating of PEDOT:PSS solution to the electrode contacts (B3), corresponding to the respective schematics in (A). Scale bar represents 200µm. C) Use of Parylene C as encapsulation layers allows conformal fitting of the device to the cortical surface. Scale bar represents 1cm. D) Electrochemical impedance spectroscopy for 20µm electrode device.

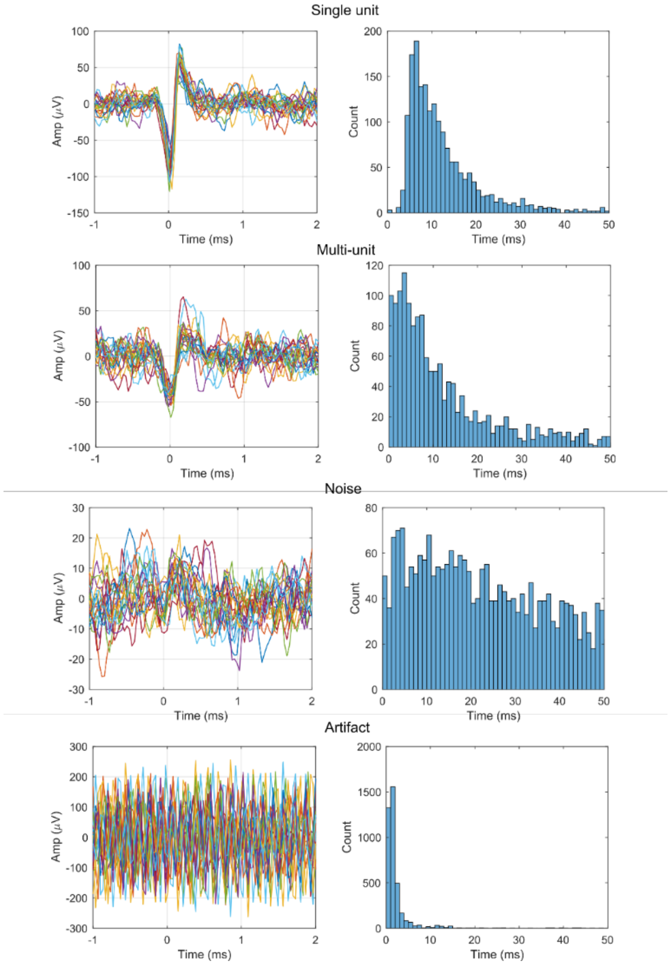

S-Figure 4: Examples of unit labeling. The four possible labels are shown from top to bottom: single unit, multi-unit, noise and artifact. On the left are 50 snippets from that cluster sampled uniformly over the recordings. On the right are inter-spike-interval histograms showing the number of spikes that occur within a certain period ranging from 0 to 50 ms after all spikes occurred.

S-Table 1. Stimulus evoked response statistics.

	**Surface (n = 11 out of 23)**	**Depth (n = 23 out of 46)**	**P-Value**	**Test**
Correlation	0.55	0.65	0.19	Rank sum
Effect Size	0.51	0.62	0.91	Rank sum
Latency	65 ms	30 ms	0.64	Rank sum

S-Table 2: **Number of single units significantly correlated with auditory onset for each subject**

	Depth	Surface
**b1061**	7	6
**b1047**	16	0
**b1067**	0	2
**b1114**	0	1
**b1107**	0	0
**b1159**	0	2
